# Repurposing of Glycine-Rich Proteins in Abiotic and Biotic Stresses in the Lone-Star Tick (*Amblyomma americanum*)

**DOI:** 10.3389/fphys.2019.00744

**Published:** 2019-06-18

**Authors:** Rebekah Bullard, Surendra Raj Sharma, Pradipta Kumar Das, Sarah E. Morgan, Shahid Karim

**Affiliations:** ^1^Department of Cell and Molecular Biology, School of Biological, Environmental and Earth Sciences, The University of Southern Mississippi, Hattiesburg, MS, United States; ^2^Department of Pathobiological Sciences, Louisiana State University, Baton Rouge, LA, United States; ^3^School of Polymer Science and Engineering, The University of Southern Mississippi, Hattiesburg, MS, United States

**Keywords:** ticks, cement proteins, glycine-rich proteins, salivary glands, *Amblyomma americanum*

## Abstract

Tick feeding requires the secretion of a huge number of pharmacologically dynamic proteins and other molecules which are vital for the formation of the cement cone, the establishment of the blood pool and to counter against the host immune response. Glycine-rich proteins (GRP) are found in many organisms and can function in a variety of cellular processes and structures. The functional characterization of the GRPs in the tick salivary glands has not been elucidated. GRPs have been found to play a role in the formation of the cement cone; however, new evidence suggests repurposing of GRPs in the tick physiology. In this study, an RNA interference approach was utilized to silence two glycine-rich protein genes expressed in early phase of tick feeding to determine their functional role in tick hematophagy, cement cone structure, and microbial homeostasis within the tick host. Additionally, the transcriptional regulation of GRPs was determined after exposure to biotic and abiotic stresses including cold and hot temperature, injury, and oxidative stress. This caused a significant up-regulation of AamerSigP-34358, Aam-40766, AamerSigP-39259, and Aam-36909. Our results suggest ticks repurpose these proteins and further functional characterization of GRPs may help to design novel molecular strategies to disrupt the homeostasis and the pathogen transmission.

## Introduction

The Lone-Star tick *Amblyomma americanum* is of significant health concern in the United States, given its expanding geographic range and vector-competence for diseases such as by *Ehrlichia chaffeensis, Borrelia lonestari, Ehrlichia ewingii, Francisella tularensis, Theileria cervi* and heartland virus (Childs and Paddock, 2003; Goddard and Varela-Stokes, 2009). *A. americanum* has recently been associated with delayed anaphylaxis to red meat and is the first recorded example of an ectoparasite causing food allergy in the United States (Commins et al., 2011; Crispell et al., 2019). The bites from *A. americanum* are causing this unusual allergic reaction to meat (Platts-Mills and Commins, 2013).

Ticks are the most successful group of organisms and have developed the hematophagous trait to feed on vertebrate blood. During the attachment phase, the tick must establish a firm attachment by secreting multiple pharmacologically active proteins and other compounds in its saliva. Some of these compounds solidify once inside the host skin to form a proteinaceous matrix called the cement cone (Bishop et al., 2002; Bullard R. et al., 2016). This cement cone protects the hypostome (mouth part) while also anchoring the tick into the host dermis. Ticks of both long (Longirostrata) and short (Brevirostrata) mouthpart species secrete this proteinaceous matrix. Analysis of multiple adhesives from various species of glue-producing insects showed a prevalence of glycine in many of the proteins identified. Of all the adhesives tested, each species contained at least one protein which was classified as glycine rich, with 11–36% glycine content (27/33 proteins). Other amino acids also overrepresented include serine (12–33% in 17 proteins) and alanine (12–17% in 2 proteins) (Zhang et al., 2013).

Saliva molecules interact with the host immune system to assist the tick in establishing a liquid blood pool. Previous data have confirmed the proteins secreted through the saliva are not constituently expressed throughout the entirety of the blood meal, but are rather differentially expressed (Karim et al., 2011; Karim and Ribeiro, 2015). These proteins are responsible for mediating the host response by preventing clot formation, wound healing, immune system activation, and inflammatory cascades (Childs and Paddock, 2003). In addition to the modulation of the host immune system, some of the secreted proteins accumulate around the tick mouthparts and harden to form a cement cone which has previously been described in detail from our lab (Bullard R. et al., 2016). Proteomic analysis of these cones have identified multiple protein families such as GRPs, protease inhibitors, mucins and various uncharacterized cement cone proteins among these GRPs are predominant (Bullard R. et al., 2016; Bullard R.L. et al., 2016). One class of proteins found in the cement cone, GRP, has documented roles of physiological functions and structural characteristics in a variety of organisms (Zottich et al., 2013; Xu et al., 2014; Bullard R. et al., 2016).

GRPs are known to be a major structural component of spider silk (Winkler and Kaplan, 2000; Xia et al., 2010; Tokareva et al., 2013), insect cuticles (Zhang et al., 2008), and plant cell walls (Mousavi and Hotta, 2005). GRPs have also been implicated in anti-microbial activity (Wang et al., 2008), anti-freeze functions (Graham and Davies, 2005; Mok et al., 2010), RNA-binding (Kim et al., 2007), and anti-platelet aggregation (Schemmer et al., 2013). Starvation responsive, injury induced response, and defense against microbes and predators have been lined with the GRPs expression in insect hemolymph (Baba et al., 1987; Taniai et al., 2014; Yi et al., 2014; Pentzold et al., 2016).

It is currently unknown if the GRPs which are upregulated during the stress response play a role in mediating the stress directly or indirectly by interfering with the gene expression. Our work on the transcriptome analysis of *A. americanum* salivary glands identified various differentially and constitutively expressed GRPs, among which 14 glycine-rich proteins contained signal peptides, and 24 lacked signal peptides (Karim and Ribeiro, 2015). In this study, a sample of tick GRPs are analyzed to identify the types of glycine repeats found within the sequence. The transcripts of two GRPs up-regulated during early tick feeding are depleted using RNA interference to examine their functional role in tick hematophagy. The transcriptional expression of these GRPs was also determined in ticks with abiotic and biotic stresses. After evaluation of the feeding phenotypes of the knockdown ticks, further analysis of GRP expression is performed to identify functions in microbial maintenance and the tick’s response to stressful stimuli.

## Materials and Methods

### Ethics Statement

All animal experiments were carried out in strict accordance with recommendations in the Guide for the Care and Use of Laboratory Animals of the National Institutes of Health, United States. The protocol of tick feeding on sheep (#15101501.1) was approved by the Institutional Animal Care and Use Committee of University of Southern Mississippi.

### Materials

All common laboratory supplies, and chemicals were purchased from Sigma-Aldrich (St. Louis, MO, United States), Fisher Scientific (Grand Island, NY, United States), or Bio-Rad (Hercules, CA, United States) unless otherwise specified.

### Bioinformatics Analysis

Coding sequences of GRPs were obtained from *A. americanum* transcriptome study (Karim and Ribeiro, 2015) and various biological databases. Sequences were aligned using muscle alignment tool^1^ and graphically presented via Snapegene viewer.

### Ticks and Tissue Dissection

Ticks were purchased from the Oklahoma State University Tick Rearing Facility. Adult unfed male and female *A. americanum* were kept according to standard practices (Patrick and Hair, 1975) at room temperature (25°C) with approximately 90% relative humidity for a photoperiod of 14 h light/10 h dark. Ticks were infested on a sheep and partially blood-fed females were pulled after 5 days of post-infestation to obtain tissues. The blood-fed female *A. americanum* were dissected within 4 h of removal and collection from the sheep. Tick salivary glands were dissected as described previously (Karim and Ribeiro, 2015). Salivary gland tissues were pooled and stored in RNAlater (Life Technologies, Carlsbad, CA, United States) at -80°C until further use.

### Transcriptional Gene Expression Analysis

#### RNA Isolation and cDNA Synthesis

The methods to extract total RNA and cDNA synthesis were conducted as described previously (Bullard R.L. et al., 2016). RNA was extracted from the pooled-dissected tick salivary glands and cDNA was synthesized from these samples to determine the transcriptional expression of GRPs. Briefly, frozen tick tissues were placed on ice to thaw and RNAlater was carefully removed with precision pipetting. RNA was isolated from the time point pooled salivary glands using illustra RNAspin Mini kit (GE Healthcare Life sciences) protocols. RNA concentration was measured using a NanoDrop spectrophotometer and stored at -80°C or used immediately. To synthesize cDNA, 2 μg of RNA were reverse transcribed using the iScript cDNA synthesis kit (Bio-Rad). The reverse transcription reaction was then set-up in a Bio-Rad thermocycler under the following conditions: 5 min at 25°C, 30 min at 42°C, 5 min at 85°C, and hold at 10°C. The resultant cDNA was diluted to a working concentration of 25 ng/μL with nuclease-free water and stored at -20°C until used (Bullard R.L. et al., 2016).

#### Reverse Transcription Quantitative PCR (RT-qPCR) Assay

A list of all genes tested in this study is provided in Supplementary Table S1. The transcript level of GRPs was quantified by a RT-qPCR assay as described earlier (Bullard R.L. et al., 2016). Briefly, 50 ng of cDNA was used in a total of 20 μL reaction using SYBR Green supermix with 300 nM of each gene specific primer. The samples were subjected to the following thermocycling conditions: 95°C for 30 s; 35 cycles of 95°C for 5 s and 60°C for 30 s with a fluorescence reading after each cycle; followed by a melt curve from 65 to 95°C in 0.5°C increments. Each reaction was performed in triplicate along with no template controls. Gene expression was normalized using ubiquitin as the reference gene and compared against treatment control.

#### DsRNA Synthesis and Tick Injections

The gene of interest was amplified using gene specific primers and purified using the QIAquick PCR Purification Kit (QIAGEN, Germany). Gene specific T7 promoter sequences were added to the 5′ and 3′ end of the purified product using PCR and were purified. The purified T7 PCR products was confirmed by sequencing and transcribed into dsRNA using the T7 Quick High Yield RNA Synthesis Kit (New England Biolabs, Ipswich, MA, United States). The dsRNA produced was purified via ethanol precipitation and the concentration was measured using a NanoDrop spectrophotometer and was analyzed on a 2% Agarose gel. Individual unfed female ticks were injected with irrelevant (GFP dsRNA) and target gene dsRNAs (AamersigP-41539, and Aam-40766) using a 31-gauge needle to a final concentration of 500 ng as described previously (Bullard R.L. et al., 2016). Ticks were kept overnight at 37°C to determine trauma/death related to microinjections. Next day ticks are infested on sheep. The ticks were infested on a sheep the next day. Attachment was monitored daily and photographed. Ticks attached within 24 h of infestation were considered attached and monitored until repletion. Partially blood-fed ticks (5 days post-infestation) were pulled and dissected for gene expression analysis.

### Quantification of Total Bacterial Load

The total bacterial load in tick tissues was determined using the method described previously (Narasimhan et al., 2014; Budachetri and Karim, 2015). Briefly, a 25 μl volume reaction mixture contained 25 ng of tissue cDNA, 200 (μM 16S RNA gene primer and iTaq Universal SYBR Green Supermix (Bio-Rad) followed by qPCR assay using following conditions: 94°C for 5 min followed by 35 cycles at 94°C for 30 s, 60°C for 30 s, and 72°C for 30 s. A standard curve was used to determine the copy number of each gene. The bacterial copy number was normalized against *A. americanum* ubiquitin copy number in control tissues and gene silenced tick. All samples were run in triplicate.

### Stress Exposure

#### Cold Stress

Supplementary Figure S1 illustrates the approaches utilized to determine the stress induced expression of glycine-rich proteins at organismal and tissue level. A total of 15 unfed female ticks were incubated at 4°C in an incubator with 90% relative humidity and a photoperiod of 14 h light/10 h dark cycle for a month to mimic winter like conditions. GRP gene expression was determined in the whole tick and dissected tissues. A total of 3 individual ticks were crushed to extract total RNA, while five pairs of salivary glands were pooled for the expression studies.

#### Heat Stress

A total of 15 unfed female adult ticks were kept at 40°C in an incubator with 42% relative humidity and a photoperiod of 14 h light/10 h dark cycle for 1 week. The expression of selected GRP genes was measured at both tissue and organismal levels as described above.

#### Injury Stress

Fifteen female ticks were injured by piercing the cuticle or pulling a hind leg from the tick. The ticks were placed in a 25°C incubator with 90% relative humidity maintaining photoperiod of 14 h light/10 h dark cycle to recover from the injury for 1 week. GRP expression was measured in both pooled salivary gland tissues (from five ticks) and in individually crushed whole ticks.

#### Oxidative Stress

Fifteen female ticks were injected with 10 mM Paraquat to induce a high oxidative stress environment in the tick tissues and allowed to recover over a 48-h period in 25 (°C incubator with 90% relative humidity maintaining photoperiod of 14 h light/10 h dark cycle. GRP expression was measured in both dissected salivary glands (pooled salivary glands from five ticks) and in crushed whole ticks (three individual ticks). In order to control for any transcriptional changes due to the injection, data from the injury stress exposed ticks were used for normalization.

### Atomic Force Microscopy, Quantitative Nanoscale Mechanical Characterization (AFM-QNM) of the Tick Cement Cone

The cones from the *in vivo* fed ticks and from the artificially membrane fed ticks were sectioned along their lengths via cryo-microtoming. The thickness of each section was 20 (μm. The sections were then analyzed via AFM-QNM. AFM-QNM was performed with a Dimension Icon (Bruker) instrument in tapping mode. Silicon nitride probes (RTESP from Bruker) with a typical resonance frequency of 324–358 kHz, spring constant of 20–80 N/m, length of 115–135 μm, and tip radius of 8 nm were utilized for the imaging. A relative calibration method using a standard polystyrene film of 2.7 GPa (from Bruker) was utilized. The AFM images were captured at a scan rate of 1 Hz and 256 × 256 pixels of data points were collected. Images were taken at different locations (at least three) across the surface. NanoScope 5.30r2 software was used to capture the images. The height images were analyzed via NanoScope Analysis 1.5 (Bruker) image processing software.

### Data Analysis

All data are expressed as mean (SEM unless otherwise stated. Statistical significance between the two experimental groups or their respective controls was determined by the *t*-test using Graph Pad prism 7 (La Jolla, CA, United States). P-values of <0.05 were considered significant. Transcriptional expression levels were determined using Bio-Rad software (Bio-Rad CFX MANAGER v.3.1), and the expression values were considered significant if the P-value was 0.05 when compared with the control.

## Results

### Bioinformatic Analysis of GRP Sequences

GRPs are characterized purely by the overabundance of glycine in the protein’s sequence. This complicates typical bioinformatic analyses which would predict structure and function by comparing the unknown protein’s sequence to other well described proteins. GRP sequences provide little information since they are not classified based on structural and functional aspects. Sequence identity is insignificant as the sheer number of glycine residues account for most of the homologous residues. To determine if the GRPs selected from the *A. americanum* sialotranscriptome (Karim and Ribeiro, 2015) could be grouped, the tripeptide and penta-peptide repeats commonly found in GRPs were used as a way to group the proteins (Table 1). When compared in this way, a possible pattern begins to emerge. GRPs with fewer amino acids typically have the GGX repeat whereas proteins with more than 200 amino acids have a near equal mixture of GGX and GXG repeats. While GXXXG repeats are found in these proteins, they are not more abundant than the tripeptide repeats listed. Interestingly, a scanning for motif identification of all nine GRPs in Prosite, only showed the presence of a motif in AamerSigP-34358 (PISGGSGGVRLPGQSGSKPG: T RNA ligase signature motif) (Table 1). Multiple sequence alignment of all selected nine GRP amino acid sequences only showed GGX and GXG repeats (Supplementary Figure S2). This pattern does not hold true when looking at GRPs from other organisms or even other ticks of the same species, so this information may not be a usable criterion for identifying GRP classes.

**Table 1:** Protein characteristics and tripeptide repeats of nine AaGRPS.

Gene ID	Number of amino acids	Molecularweight (kDa)	Noteworthyamino acidprevalence	Noteworthy amino acid repeats/motif	GRPs class based on amino acid repeats
Aam-41235 (GBZX01001012.1)	107	10.0	22.4%Gly 22.4% Ser 15.0% Ala	GGX	Class I
AamerSigP-34358 (GBZX01000232.1)	205	18.7	30.7% Gly 17.1% Ser 13.2% Pro	GGX/GXG/ PISGGSGGVRLP GQSGSKPG	Class I /III
Aam-40766 (GBZX01000067.1)	310	29.4	27.7% Gly 19% Ser 11% Ala	GGX/GXG	Class I/III
AamerSigP-39259	54	51.7	22.2% Gly 16.7% Ser 13.0% Ala	GGX/GXG	Class I/III
AamerSigP-41913 (GBZX01000836.1)	116	11.8	19.8% Gly	GGX	Class I
AamerSigP-41539 (GBZX01000853.1)	116	11.6	24.1% Gly 13.8% Ala 10.3% Ser	GXG/GGX	Class I/III
Aam-41540 (GBZX01001942.1)	222	22.6	20.3% Gly 13.1% Ala	GGX	Class I
Aam-36909 (GBZX01000052.1)	325	UD	29.2% Gly 20.6% Ser	GGX/GXG	Class I/III
Aam-3099 (GBZX01000254.1)	202	20.0	23.3% Gly 15.3% Ala	N/A	N/A

*GRP classes are categorized based on presence of amino acid sequence repeats.*

### Expression of GRPs After the Depletion of a GRP Transcript and Cement Cone Analysis

**FIGURE 1:**
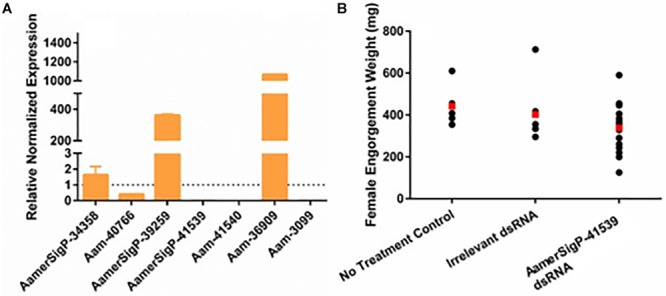
RNA interference-based silencing of AamerSigP-41539 (glycine-rich protein gene) in the lone-star tick (*Amblyomma americanum*). **(A)** Compensatory transcriptional expression of selected grp genes was determined in AamerSigP-41539 silenced partially blood-fed tick salivary glands. **(B)** Mass of replete or forcibly removed 12 days fed female ticks. Ubiquitin was used as a housekeeping gene to normalize the transcriptional expression.

The injection of dsRNA-AamerSigP-41539 (GXG/GGX) effectively depleted AamerSigP-41539 transcripts by >95% (data not shown). Each of the remaining GRPs were also analyzed to determine any off-target or compensatory effects (Figure 1A). Although the GRP transcript was significantly reduced, there was no measurable change in the ability of the tick to attach to the host, maintain a firm attachment, or ability to feed successfully (Figure 1B). Two GRPs, AamerSigP-39259 (GGX/GXG) and Aam-36909 (GGX/GXG), are over expressed when AamerSigP-41539 is depleted (Figure 1A). Interestingly, the depletion of AamerSigP-41539 also results in the depletion of Aam-41540 (GGX) and Aam-3099 but there is no significant change in AamerSig-34358 or Aam-40766 (Figure 1A).

**FIGURE 2:**
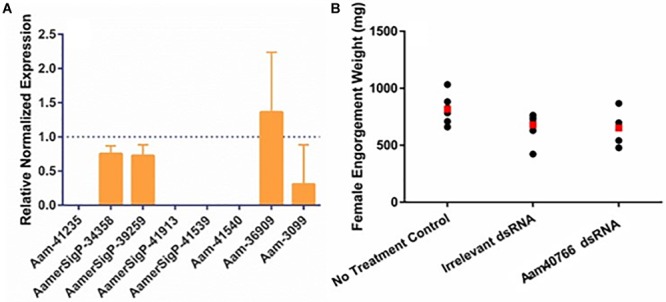
RNA-interference based gene silencing of Aam-40766 (glycine-rich protein gene) in the lone-star tick (*A. americanum*). **(A)** Transcriptional expression of Aam-40766 and other glycine-rich proteins in the gene silenced partially blood-fed tick tissues. **(B)** Mass of replete or forcibly removed 12 days fed female ticks. Ubiquitin was used as a housekeeping gene to normalize the transcriptional expression.

Similar methods were used to elucidate the impact of gene silencing of an additional GRP, Aam-40766 (GGX/GXG). The depletion of Aam-40766 transcripts were verified using qRT-PCR and impact of gene silencing was monitored as feeding progressed. As seen with AamerSigP-41539, the depletion of Aam-40766 yielded no significant changes in attachment or engorgement weight (Figure 2B). The expression of the other GRPs was measured to identify compensatory mechanisms (Figure 2A). The depletion of Aam-40766 resulted in the reduction of five other GRPs (Aam-41235, AamerSigP-41913, AamerSigP-41539, Aam-41540, and Aam-3099). The other GRPs tested showed no change in expression levels.

**FIGURE 3:**
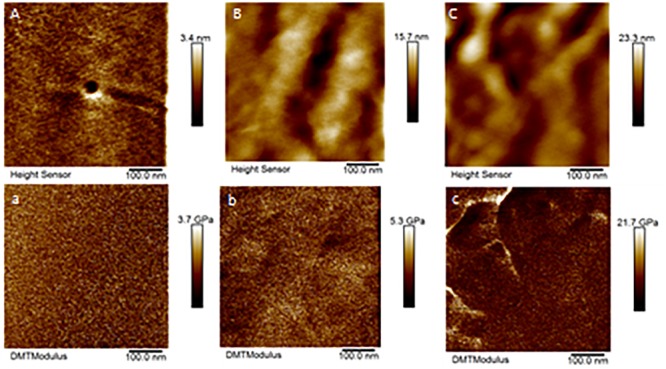
AFM-QNM images of the cement cone obtained from a naturally fed tick. **(A,a), (B,b), (C,c)** are the height and modulus images of the section 1, 2, and 3 of the cement cones, respectively. An average modulus of 2.9, 4.6, 7.1 GPa was obtained for the section 1, 2, and 3, respectively.

**FIGURE 4:**
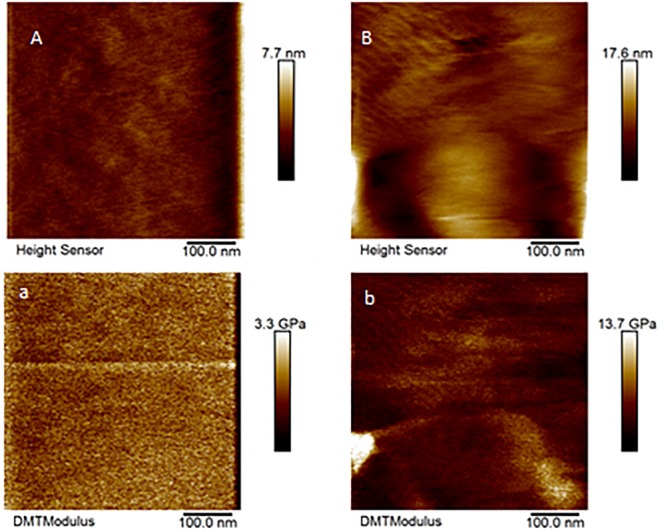
AFM-QNM images of the cement cone obtained from an Aam-41539 silenced tick. **(A,a)**, and **(B,b)** are the height and modulus images of the section 1, and 2 of the cement cones, respectively. An average modulus of 2.5, and 6.2 GPa was obtained for the section 1, and 2, respectively.

Only a few cones from Aa-41539 depleted ticks and irrelevant double stranded RNA injected ticks were collected with cement cones attached at the tick mouthparts. However, it is important to note that of all the ticks fed, less than 10 cones were collected which made it difficult to determine whether the lack of cement cones from knocked-down ticks is due to a change in phenotype or just a complication of cement cone retrieval. AFM-QNM analysis of cone (Figure 3) recovered from control (irrelevant double stranded RNA injected) ticks showed that the modulus of the cone varies in different regions of cone. More specifically, the modulus of the cone decreases (2.9; 4.6; 7.1 GPa) as the cement penetrates deep into the host skin. Similarly, AFM-QNM analysis of the cement cone obtained from an AamSigP-41539 (Figure 4) depleted tick showed smaller than average modulus (2.5 and 6.2 GPa) in comparison to related section of cones (2.9 and 7.1 GPa) from control ticks.

### Impact of GRP Silencing on Total Microbial Load

**FIGURE 5:**
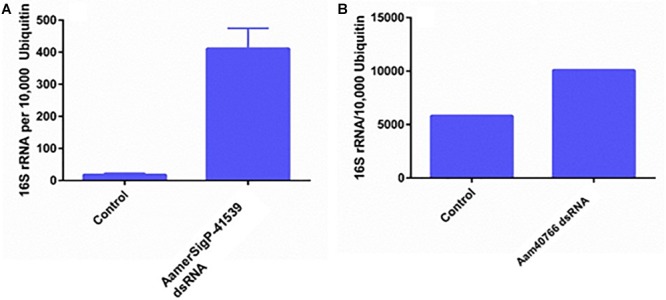
Total bacterial load in GRP silenced tick tissues. The ticks from both irrelevant dsRNA (GFP) and target genes were blood feed and partially fed ticks were removed from the host past 5 days infestation. Within 2 h of tick removal from the hosts, the ticks were dissected to isolate salivary glands and stored in RNAlater before RNA extraction and cDNA synthesis. Total bacterial loads were estimated by qPCR and reference to ubiquitin in the tick salivary glands. **(A)** Changes in 16S rRNA abundance after depletion of AamerSigP-41539. **(B)** Changes in 16S rRNA abundance after depletion of Aam-40766.

GRPs have been implicated in a variety of functional roles throughout the animal and plant world. Given that no measurable difference was observed in attachment or feeding in the GRP depleted ticks, it is possible that GRPs AamerSigP-41539 and Aam-40766 may play a role in pharmacological characteristics of tick saliva. To determine if tick GRPs may play a role in maintaining bacterial communities, the total bacterial load was calculated by measuring 16S rRNA in the tissue. In the irrelevant dsRNA injected ticks, after 5 days of feeding, there were approximately 20 16S rRNA molecules for every 10,000 tick Ubiquitin (Figure 5A). However, in the case of AamerSigP-41539 deficient ticks, this increases to 400 16S rRNA molecules for every 10,000 ubiquitin. This 20-fold increase signifies that AamerSigP-41539 is at least partially responsible for the maintaining of microbial homeostasis within the tick salivary glands (Figure 5A). The exact mechanism of this role is yet to be determined. Additional studies on this protein, it’s mechanism and how it affects microbial growth requires further investigation. In contrast to the significant bacterial growth in AamerSigP-41539 depleted ticks, when Aam-40766 is knocked down, the change in 16S rRNA is only 2-fold (Figure 3B). It should be noted that the AamerSigP-41539 ticks were partially fed for 5 days while the Aam-40766 ticks were partially fed for 8 days. This could have an effect on the magnitude difference between the two studies. It was noticed during data analysis of all datasets (including those not shown here) that GRPs were upregulated even in non-relevant dsRNA injections such as GFP. During RNAi, the ticks are subjected to injections and high humidity heat conditions to recover from injury trauma. Previous research in plants has shown the increased presence of GRPs during wound healing. A tick GRP responsible for wound healing would explain these results. A look into the expression of GRPs during stress conditions and other known GRP functions were then performed.

### Effect of Abiotic Stress on GRP Transcript Levels

**FIGURE 6:**
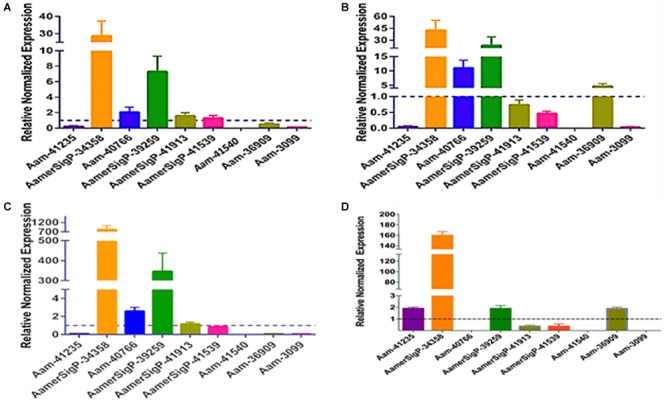
Transcriptional expression of tick GRP genes in unfed ticks after stress exposure. Quantitative reverse transcriptase PCR (qRt-PCR) was used to determine the transcriptional expression levels of tick GRPs. **(A)** cold temperature, **(B)** hot/humid temperature, **(C)** injury, and **(D)** oxidative stress. The expression levels in tick control samples were set to 1, as represented by dashed lines. Ubiquitin was used as a housekeeping gene to normalize the transcriptional expression.

To further identify potential GRP functions within the salivary glands, ticks were exposed to low temperatures, high temperatures, injury, and oxidative stress to identify stress related GRPs (Figure 6). The GRP expression profile of tick tissues after cold exposure shows the differential expression of many GRPs. The cold temperature presumably decreases the metabolic rate of the tick which down regulates many proteins (Figure 6A). The decreased expression of Aam-41235 (4-fold), Aam-36909 (2-fold), Aam-3099 (9-fold), and the complete depletion of Aam-41540 transcripts are conceivably due to this decrease in metabolic rate or the GRP is not expressed in the unfed time stages. The low temperature, however, does cause an increase in AamerSigP-34358 (29-fold), Aam-40766 (2-fold), and AamerSigP-39259 (7-fold). An increase in the temperature also affects the GRP expression profile in the whole tick samples. When the ticks are exposed to high temperatures (Figure 6B), there is a decrease in Aam-41235 (20-fold), AamerSigP-41539 (2-fold), and Aam-3099 (31-fold). But there is an increase in many of the other GRPs including AamerSigP-34358 (43-fold), Aam-40766 (11-fold), AamerSigP-39259 (23-fold), and Aam-36909 (5-fold). The GRP expression of injured ticks was also measured. These tick samples had a decrease in GRPs such as Aam-41235 (10-fold), Aam-36909 (11-fold), and Aam-3099 (13-fold). The expression of AamerSigP-34358 and AamerSigP-39259 were again upregulated like the other stress conditions; however, the fold change is much higher (83-, and 347-fold, respectively). Similarly, oxidative stress in the whole tick showed upregulation of signal peptides namely AmerSigP-34358 (160 fold); AamerSigP-39259 (2 fold). Two GRPs such as Aam-41235 and Aam-36909 were found to be upregulated by two-fold while hypothetical secreted peptides AamerSigp-41539 and AamerSigp-41913 were found to be down-regulated; however, there was no expression of Aam-41540, Aam-40766, and Aam-3099 under oxidative stress in the whole tick.

**FIGURE 7:**
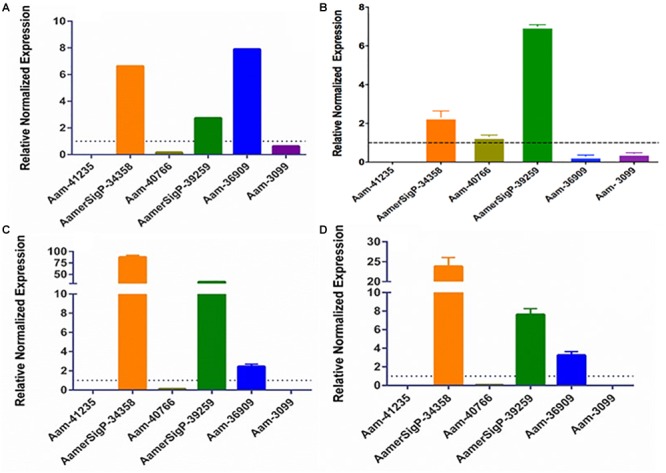
Transcriptional expression of tick GRP genes in unfed tick salivary glands after stress exposure. Quantitative reverse transcriptase PCR (qRt-PCR) was used to determine the transcriptional expression levels of tick GRPs. **(A)** cold temperature, **(B)** hot/humid temperature, **(C)** injury, and **(D)** oxidative stress. The expression levels in tick control samples were set to 1, as represented by dashed lines. Ubiquitin was used as a housekeeping gene to normalize the transcriptional expression.

The gene expression in the salivary glands show a similar change when exposed to stress (Figure 7). The salivary glands of ticks exposed to the low temperature (Figure 5A) have an increase of AamerSigP-34358 (6.6-fold), AamerSigP-39259 (3-fold), and Aam-36909 (8-fold). There is a significant decrease in Aam-40766 (6-fold), however, no change is seen in Aam-3099. Aam-41235 did not amplify in the salivary glands, and AamerSigP-41913, AamerSigP-41539, and Aam-41540 were not tested due to limited sample availability. Under heat stress there was upregulation of two signal peptides, AamerSigP-34358 and AamerSigP-39259 by three-fold and seven-fold, respectively. However, protein Aam-40766 was slightly upregulated while other proteins such as Aam-36909 and Aam-3099 was downregulated by eight-fold and seven-fold, respectively while there was no expression of Aam-41539.

Similar results are seen in salivary glands after an injury has occurred to the tick (Figure 7C). A decrease in Aam-40766 (8-fold) and the non-amplification of Aam-41235 and Aam-3099 show a change in the salivary glands from preparing for attachment and blood feeding to dealing with the trauma of the injury. Although the salivary glands are not directly injured by the removal of one of the hind legs, there is an increase in AamerSigP-34358 (88-fold), AamerSigP-39259 (34-fold), and a slight increase of Aam-36909 (2.4-fold). As the tick is feeding, it encounters a wide range of reactive oxygen species, which can cause oxidative stress in the tick. The GRP expression after injection with paraquat is similar to that of cold and injured ticks (Figure 7D). There is no amplification in Aam-41235 or Aam-3099, a decrease in Aam-40766 (17-fold) and an increase in AamerSigP-34358 (24-fold), AamerSigP-39259 (8-fold), and Aam-3099 (3-fold).

## Discussion

The functional characterization of novel molecules regulating diverse physiological responses in ticks, is very important for targeted control of ticks. To catalog the salivary transcripts, we have carried out a comprehensive RNA-Seq analysis of the *A. americanum* salivary glands (Karim and Ribeiro, 2015). From the sialotranscriptome data, identification of several GRPs prompted us to characterize their functional role in tick hematophagy. Existing literature indicated that GRPs are responsible for mediating the host response by preventing clot formation, wound healing, immune system activation, and inflammatory cascades (Francischetti et al., 2009). However, in many insects and arthropods, GRPs play a significant structural role. From spider silk to barnacle glue, GRPs are necessary for adhesion and strength. The selection of both Aam-40766 and AamerSigP-41539 was based on their up-regulation during early phase of tick feeding on the host as reported in our published work (Bullard R.L. et al., 2016). It was hypothesized that the reduction of GRP transcripts up-regulated during early phase of tick feeding would interfere with cement cone formation thereby, making attachment difficult both at the initial attachment and throughout the prolonged blood meal. This was not the case as well as no change in blood meal uptake was observed (Figure 1B, 2B). This lack of lethal phenotypic change could be due to prepackaged GRPs synthesized before the injection of dsRNA. The existence of these proteins prior to the initiation of feeding would allow the tick to immediately utilize these proteins to establish the bite site and begin cement cone formation. Previously in this research group, we have shown that transcript depletion alone was not sufficient to change the feeding phenotype but when combined with protein inhibitors, a lethal phenotype was observed (Kumar et al., 2016). Hence, this indicates that RNAi alone might not be the perfect tool to study genes involved in attachment on the host, which are expressed early tick feeding stages.

**FIGURE 8:**
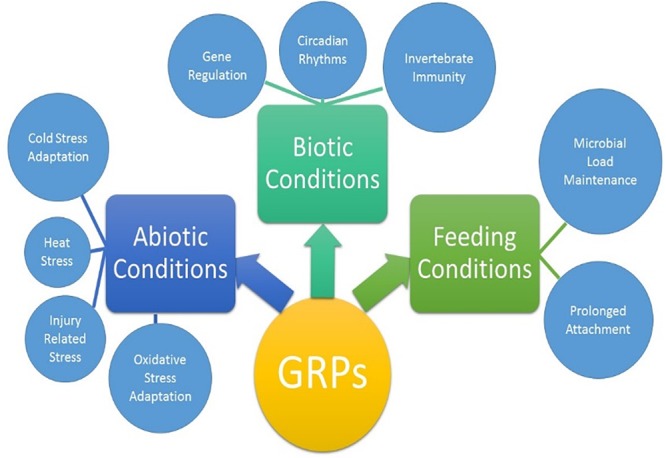
Proposed biological activities of GRPs in tick biology. The extent of which each GRP participates in all listed physiological processes are not yet clear.

Ticks must use their barbed mouthparts (hypostome) to pierce deeply into the host’s dermis (skin), then incase the hypostome in a narrow secreted cement cone. In addition to maintaining a stealthy but secure attachment, the cement cone provides a conducive environment for the injection of pathogenic microbes into the host. Cement cones have been shown to contain a number of GRPs, which display significant structural and functional heterogeneity across the animal and plant kingdoms, suggesting their involvement in multiple physiological processes (Figure 8). The AFM-QNM result of related microtome section of cement cone indicated significant variation of their modulus. Cement which is close to the epidermis of the host skin exhibited higher modulus and the cement formed deep inside the host skin had lower modulus among control and Aam-41539 silenced ticks which indicates that of Aam-41539 might have vital role in proper cone development. Since Aam-41539 is highly expressed in early feeding stage along with the slight changes seen in cone development and homologous peptide sequence have been identified in previous cone proteome analysis it can be expected that this GRP might have direct or indirect roles in the development of cone (Karim and Ribeiro, 2015; Bullard R. et al., 2016; Hollmann et al., 2018). While removing the ticks from the host, care was taken to ensure maximum cone retrieval; however, it was not possible to collect cones from each test condition at the same point of feeding. This is a complication of this area of research and therefore cannot be considered indicative of changes in the cement cone formation without significant investigation.

The tick genome is incredibly large and contains multiple copies of structurally and functionally similar proteins. Previous data from this lab has shown these proteins are not expressed all at once or all throughout the blood meal but rather during small time frames of the blood meal (Bullard R.L. et al., 2016). This could be evolutionarily designed so that the tick is able to switch through all of the available genes to prevent detection from the host immune system. This becomes more complicated when applying this concept to GRPs. Because of the way GRPs are identified, it is possible that the nine selected nine GRPs may posses variety of functions. This would not fit into the switching hypothesis as the expression of one GRP would not compensate for another. The GRP expression in AamerSigP41539 depleted ticks, shows the upregulation of three Aa-GRPs (AamerSigP-34358, AamerSigP-39259, and Aam-36909, Figure 1A). However, when these results are compared to the gene expression in ticks suffering from injury stress, the same genes are upregulated. It is unlikely that the changes in the gene expression are a response from the tick to compensate for the loss of function from AamerSigP-41539 but rather a response to the injection injury during the delivery of the double stranded RNA.

There is a class of GRPs with antimicrobial activities in insects. Glycine rich antimicrobial peptides (GR-AMPs) are typically small peptides and can act on the microbes in a bacteriostatic fashion. Gloverins, an anti-microbial peptide isolated from *Hyalophora gloveri*, contains 18% glycine and has no significant similarities to known antimicrobial peptides. Antimicrobial peptides can range from only a few dozen residues to a few hundred as gloverins and attacins (Yi et al., 2013). Based on this criterion, the AaGRPs could serve as antimicrobial peptides. Another family of glycine rich antimicrobial peptides are plasticins from South American hylid frogs (Scorciapino et al., 2013). Plasticins are able to disrupt the membrane mimetic environments (Carlier et al., 2015)such as a cell membrane of evading pathogens.

The increase in 16S rRNA is used as a measure of bacterial growth in the salivary glands. However, each bacterial cell contains more than 1 copy of 16S rRNA and so the increase cannot be taken as a true increase of bacteria. In the case of Aam-40766 depleted ticks, there is a 2-fold increase of 16S rRNA molecules (Figure 5B). This increase could be due to an increase in bacteria or to an increase of protein production in the bacteria or a mixture of both. The 20-fold increase of 16S rRNA in AamerSigP-41539 depleted ticks (Figure 5A), however, cannot be attributed solely to an increase in protein production. This increase must be due, at least in part, to an increase in bacteria cells.

In plants, GRPs are well documented to exhibit differential expression during multiple stress conditions. In Arabidopsis, a glycine rich domain protein (*AtGRDP2*) which is expressed throughout plant development and when AtGRDP2 is functionally not present there is a decrease in plant growth (Ortega-Amaro et al., 2015). The over expression of this protein results in increased growth and increased tolerance to stress conditions such as increase salinity (Ortega-Amaro et al., 2015). GRPs have also been differentially expressed in *Bombyx mori* after periods of starvation, although the exact mechanism involved has not yet been identified (Taniai et al., 2014). The lone-star ticks have expanded their geographic range into new areas of the northern and mid-western United States (Munzon et al., 2016; Sonenshine, 2018). Range expansion of *A. americanum* presents a significant public health threat in northeastern and southern Canada (Springer et al., 2015). The northward expansion of this tick’s geographic range is consistent with climate change. This also suggests the possibility of adaptive evolution in distinct tick populations from New York, Oklahoma, and historic populations in the Carolinas (Munzon et al., 2016). To explore the role of AaGRPs in the stress response, ticks were exposed to various stress conditions such as cold temperature, hot temperature, oxidative stress, and injury (Figure 6, 7). When the gene expression of stressed salivary glands is compared to stressed whole ticks, it becomes apparent that some of the genes are differentially expressed in different tissues. This is most evident in the expression of Aam-40766 and Aam-36909. In stressed salivary glands (Figure 7), Aam-40766 is down regulated in each of the conditions. However, when the whole tick is used, Aam-40766 is upregulated which indicates there might be tissue specific regulation of Aa-40766 expression. It is also possible that this protein is utilized by other tick tissues and is not necessary for blood feeding. Contrary to this, Aam-36909 is down regulated in cold stress and injury stress of whole ticks (Figure 6) but is upregulated in both of those stress conditions in the salivary glands. Aam-36909 may play a role in a cellular process in the salivary glands which is necessary for proper function but is not present to any large extent in the other tissues. More work is required to determine the mechanism of stress mediation.

## Conclusion

Gene depletion of two GRPs by RNAi revealed that identifying the GRPs responsible for cement cone formation will be much more difficult than depleting a few proteins. The redundant nature of the tick genome allows for many levels of compensatory mechanisms. While the gene expression of GRPs after AamerSigP-41539 depletion seems to reveal one such compensatory mechanism, further investigation of the GRP functions revealed that this is a response to the injection injury not to the depletion of AamerSigP-41539. The identification of the effect of stress on GRP expression renders routine studies impossible. An attempt was made at knocking down AamerSigP-34358 which is upregulated during the early feeding as well as during stress. The injection of the double stranded RNA increased the transcripts to such a level that the “depleted” ticks contained more transcripts than the no treatment controls. Therefore, to determine the functions of GRPs, other methods must be developed which do not induce a stress on the tick.

The finding that GRPs are involved in the stress response of the ticks leads to the question of why these genes are over expressed during the feeding stages. Typically feeding is not considered a stress on the tick as it is a necessary process. A new hypothesis has emerged from the data presented here that individual GRPs may play multiple roles during different stages of the tick life cycle. The GRPs may function as antimicrobial or serve any of the other necessary functions for blood feeding when a tick is attached to the host but during times of molting, fasting, or overwintering the same GRPs may be repurposed for stress mediation. A growing body of evidence suggest that GRPs are involved in the physiological and evolutionary adaptation of organisms to abiotic and biotic stresses (Figure 8). The role of GRPs remain mostly elusive in the context of cement cone assembly, covert attachment, and cone disassembly. The diversity of functions and structural domains, together with different but specific expression patterns, indicate that this complex protein group can be implicated in numerous physiological functions (Figure 8). Ticks are uniquely adapted to variety of stress including prolonged feeding, abiotic, and biotic stresses during its life cycle. More work is required to fully elucidate the functions of the proteins, and recombinant protein expression will be key to that process. A functional characterization of GRPs may help to design novel molecular strategy to disrupt the homeostasis and hence the pathogen transmission.

## Data Availability

All datasets generated for this study are included in the manuscript and/or the Supplementary Files.

## Author Contributions

RB and SK conceived and designed the experiments. RB, SRS, PKD, and SK performed the experiments. RB, SEM, and SK analyzed the data. SEM and SK contributed reagents, materials, and analysis tools. RB, SRS, and SK wrote the manuscript. All authors have read and approved the final version of the manuscript.

## Conflict of Interest Statement

The authors declare that the research was conducted in the absence of any commercial or financial relationships that could be construed as a potential conflict of interest. The handling Editor declared a past co-authorship with one of the authors SK.

## Footnotes

1 http://www.ebi.ac.uk

## Supplementary Material

FIGURE S1 Experimental design to examine the transcriptional expression of glycine-rich proteins at organismal and tissue level after stress exposure.

FIGURE S2 Multiple sequence alignment of selected glycine-rich proteins. Sequences are highlighted based on amino acid property and conservation.

TABLE S1 List of primers used in this study.
