# Supplementary figures and images for: Repurposing of Glycine-Rich Proteins in Abiotic and Biotic Stresses in the Lone-Star Tick (Amblyomma americanum)
Source: Front Physiol. 2019 Jun 18;10:744. doi: 10.3389/fphys.2019.00744 (PMC6591454; doi:10.3389/fphys.2019.00744)

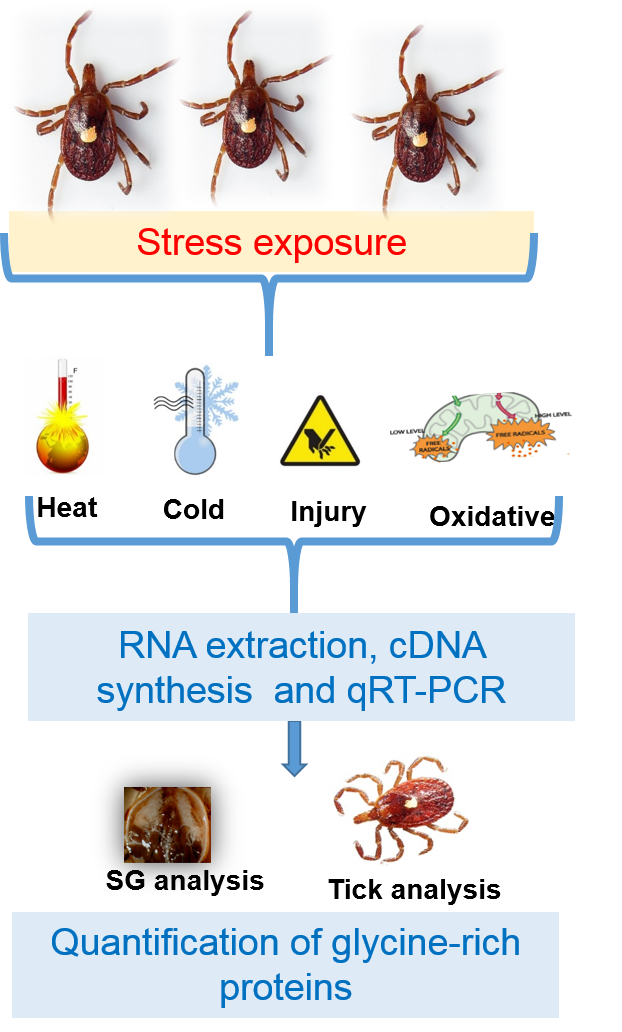

Supplement: FIGURE S1 — Experimental design to examine the transcriptional expression of glycine-rich proteins at organismal and tissue level after stress exposure. [file Image_1.TIF]

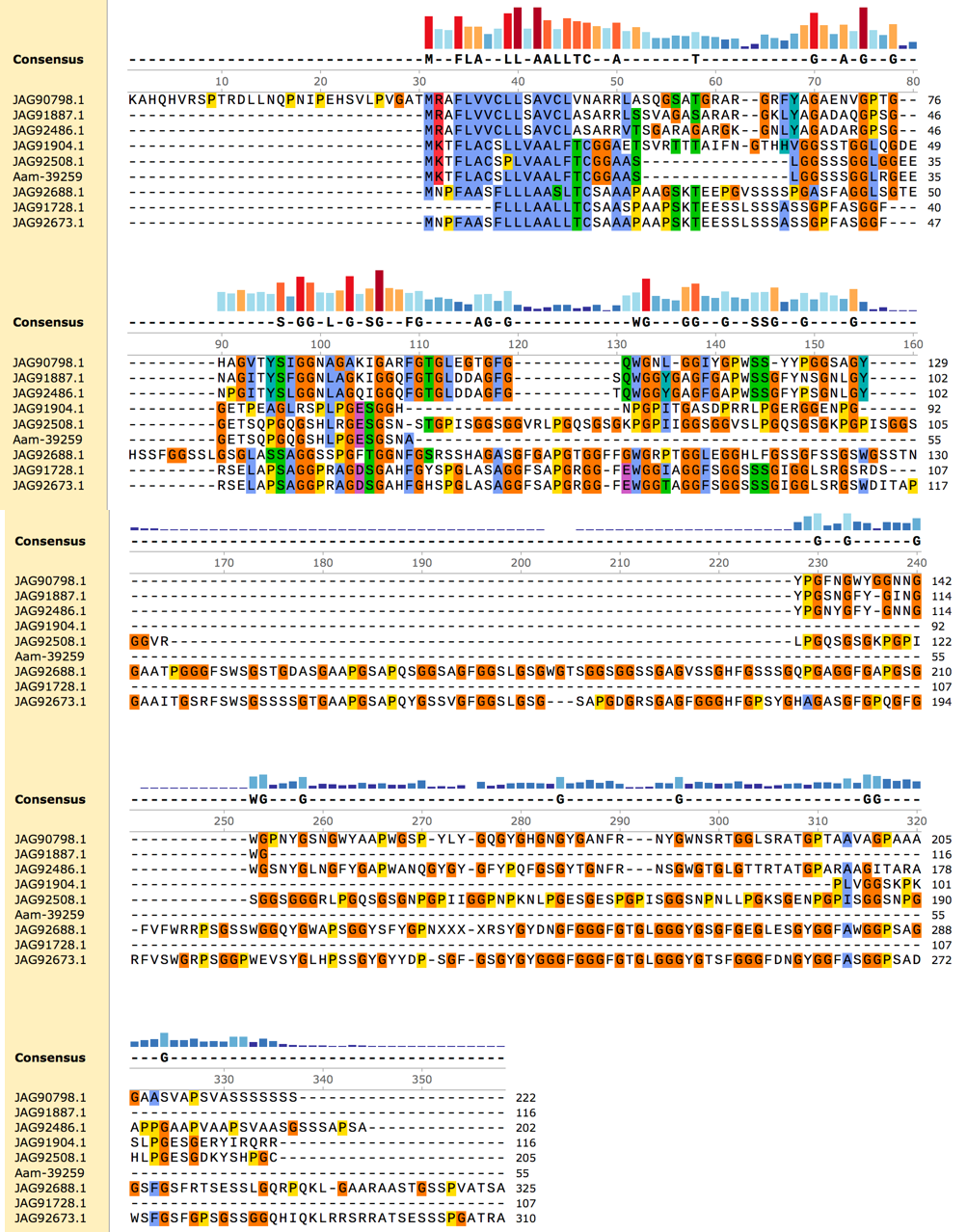

Supplement: FIGURE S2 — Multiple sequence alignment of selected glycine-rich proteins. Sequences are highlighted based on amino acid property and conservation. [file Image_2.TIF]
