# Supplementary material for: Repurposing of Glycine-Rich Proteins in Abiotic and Biotic Stresses in the Lone-Star Tick (Amblyomma americanum)
Source: Front Physiol. 2019 Jun 18;10:744. doi: 10.3389/fphys.2019.00744 (PMC6591454; doi:10.3389/fphys.2019.00744)
Supplement: TABLE S1 — List of primers used in this study. [file Table_1.docx]

**Table 1S: List of primers used in this study**

| Gene classification | Gene Name | Forward Primer 5’→3’ | Reverse Primer 3’→5’ | Amplicon size (Base pair) |
| --- | --- | --- | --- | --- |
| Housekeeping Gene | Aa Ubiquitin (GBZX01002387.1) | GCTGTCCGACTACAACATTCA | GGGTGGTGTAGTTCTTCTTCTT | 98 |
|  |  |  |  |  |
| Glycine Rich Proteins | Aam-41235 (GBZX01001012.1) | GTTGTTGGCGGCTTTGTT | GAGAGGCTGCTTTCTTCAGT | 75 |
|  | AamerSigP34358 (GBZX01000232.1) | GCGAGGAGAAAGTGGAAGTAA | ATGATGGGACCAGGCTTTC | 102 |
|  | Aam-40766 (GBZX01000067.1) | GATTTGGTGGCTCACTTGGT | AAGACCAGTGCCAAATCCAC | 100 |
|  | AamerSigP39259 | CTTTCTTGGCATGCTCCCTT | CCCTCTCAAACCACCACTAGA | 92 |
|  | AamerSigP41913 (GBZX01000836.1) | GTGGAGCTGAAACCTCTGTTAG | TCTGGAGTCTCACCTTCATCTC | 109 |
|  | AamerSigP41539 (GBZX01000853.1 | GCTAGATGACGCTGGGTTT | TCCAAGGTTTCCAGAGTTGTAG | 97 |
|  | Aam-41540 (GBZX01001942.1) | GGGAAATCTTGGCGGTATCT | GCCTCCATACCATCCGTTAAA | 94 |
|  | Aam-36909 (GBZX01000052.1) | AGCGGTGGATACAGTTTCTATG | TCCAAGACCAGTGCCAAATC | 96 |
|  | Aam-3099 (GBZX01000254.1) | GCCTTCTGTCAGCTGTATGT | CGGCGTAGAGATTACCCTTTC | 90 |
| Other Primer | 16S RNA | AGAGTTTGATCCTGGCTCA | ATGCTGCCTCCCGTAGGAGT | - |
|  | T7GFP Primer | **GAATTAATACGACTCACTATAGGGAGA**  GTCTTGTAGTTCCCGTCATCTT | **GAATTAATACGACTCACTATAGGGAGA**  AGCCAACACTTGTCACTACTT | 208 |
|  | T7AamerSigP41539  T7 Aam-40766 | **GAATTAATACGACTCACTATAGGGAGA**  GCCTTCTGTCAGCAGTATGT  **GAATTAATACGACTCACTATAGGGAGA**  GATTTGGTGGCTCACTTGG**T** | **GAATTAATACGACTCACTATAGGGAG**  ATCTTTCCTGCGAGGTTTCC  **GAATTAATACGACTCACTATAGGGAGA**  AAGACCAGTGCCAAATCCAC | 207  266 |
|  |  |  |  |  |
|  |  |  |  |  |
|  |  |  |  |  |
